# Hfq and RNase R Mediate rRNA Processing and Degradation in a Novel RNA Quality Control Process

**DOI:** 10.1128/mBio.02398-20

**Published:** 2020-10-20

**Authors:** Ricardo F. dos Santos, José M. Andrade, Joana Pissarra, Murray P. Deutscher, Cecília M. Arraiano

**Affiliations:** a Instituto de Tecnologia Química e Biológica António Xavier, Universidade Nova de Lisboa, Oeiras, Portugal; b Department of Biochemistry and Molecular Biology, Miller School of Medicine, University of Miami, Miami, Florida, USA; Institut Pasteur

**Keywords:** Hfq, RNA maturation, RNA processing, RNA quality control, RNase R, ribosomal RNA

## Abstract

RNA quality control pathways are critical for cell survival. Here, we describe a new surveillance process involved in the degradation of highly structured and stable ribosomal RNAs. The results demonstrated that the RNA chaperone Hfq and the 3′–5′ exoribonuclease R mediate the elimination of detrimental rRNA fragments and are required for the correct processing of rRNA precursors. Escherichia coli cells lacking both Hfq and RNase R accumulate a high level of 16S- and 23S-derived rRNA fragments. Hfq and RNase R were also shown to participate in the maturation of 16S and 23S rRNA precursors. This correlates with the fact that in the absence of Hfq and RNase R, there are severe ribosome assembly defects and a sharp reduction in 70S ribosome levels. Hfq and RNase R may act independently or in a complex, as protein interaction studies revealed that these RNA-binding proteins can associate. This is the first demonstration that the well-conserved Hfq and RNase R proteins act on common regulatory pathways, unraveling previously unknown mechanisms of rRNA surveillance with important consequences for translation and cell survival.

## Abstract (precis)

Quality control pathways that oversee the quality of stable RNA molecules are critical for the cell. In this work, we demonstrate, for the first time, a functional link between Hfq and RNase R in the processing and degradation of the highly structured rRNAs. These RNA-binding proteins are required for the maturation of 16S and 23S rRNAs and correct ribosome assembly. Furthermore, they participate in the degradation of rRNAs and clearance of toxic rRNA fragments from the cell. Our studies have also shown that Hfq and RNase R can form a complex. In summary, the cooperation between Hfq and RNase R in metabolic pathways of stable RNAs may represent a broader mechanism of RNA quality control, given the high conservation of these RNA-binding proteins throughout evolution.

## INTRODUCTION

Cells dispense a large amount of resources on the biosynthesis and quality control of essential translation components. In fast-growing Escherichia coli cells, most of transcription (∼80%) is devoted to rRNA synthesis, and one-fourth of the translational activity is allocated to the production of ribosomal proteins (1, 2). Therefore, the degradation of rRNA molecules is part of normal cellular metabolism, showing a growth rate dependence as longer doubling times increase the pool of degraded RNAs (3). Indeed, RNA degradation is highly important under growth-limiting conditions, in which rRNA and tRNA become repositories for nutrient recycling (4). Even under normal growth conditions, cells require surveillance mechanisms for degrading these RNAs and their by-products arising from processing and degradation. However, elimination of such stable RNAs imposes a challenge on the cell, since these molecules are usually highly structured and their accumulation is often lethal (3).

RNA quality control mechanisms targeting rRNA are critical for translation, namely, by acting as surveillance pathways for the correct assembly of ribosomes. Defective rRNA molecules that escape such control are perilous, as these may still be incorporated into 70S particles, ultimately resulting in defective ribosomes. In E. coli, a quality control mechanism that involves the endoribonuclease YbeY and the 3′–5′ exoribonuclease RNase R specifically recognizes and degrades 70S ribosomes with defective 30S subunits that are enriched in precursor 16S rRNA (5). Our previous work also demonstrated that RNase R associated with ribosomes and comigrated with the 30S ribosomal subunits (6). RNase R belongs to the RNB family of ribonucleases (7–9) that can globally impact gene expression (10), and RNase R mutants helped to elucidate the catalysis of structured RNA (11). In fact, RNase R is a unique exoribonuclease involved in various RNA surveillance pathways. RNase R has been shown (i) to be involved in the elimination of aberrant rRNAs fragments together with polynucleotide phosphorylase (PNPase) (3); (ii) to participate with RNase II in the extended degradation of ribosomes during starvation (12); (iii) to process the *tm*RNA involved in the *trans*-translation mechanism (13, 14); and (iv) to degrade non-stop mRNAs in stalled ribosomes (15). Furthermore, RNase R can affect the expression levels of r-proteins, as it was shown to degrade the mRNA encoding ribosomal protein S15 (*rpsO* gene) under conditions that favor RNA polyadenylation-dependent decay (16). The intimate relationship between RNase R and ribosomes is further underlined by the evidence that RNase R is involved in the 3′ end processing of the 16S rRNA precursor (17S rRNA) and that ribosomes regulate RNase R stability (9, 17, 18).

RNase R and the RNA-binding protein Hfq were found to interact with the same binding interface of the S12 protein from the 30S ribosomal subunit (19). Hfq is a bacterial RNA chaperone that belongs to the Sm/Lsm superfamily of proteins (20, 21). Although it is widely recognized for its ability to bind small noncoding RNAs and their mRNA targets, Hfq displays a wide spectrum of RNA substrates and protein partners (22). We have recently demonstrated that Hfq is required for the maturation of the 16S rRNA and can associate with immature 30S particles, revealing a novel role as a ribosome biogenesis factor (23). However, a functional link between RNase R and Hfq has not yet been addressed.

In this work, we describe the cooperation between Hfq and RNase R in novel rRNA surveillance pathways. Both RNA-binding proteins were found to mediate the elimination of hazardous rRNA fragments. Furthermore, the double inactivation of Hfq and RNase R strongly affects 16S and 23S rRNA maturation, which correlates with an altered ribosome profile. We show that Hfq and RNase R can associate and may act independently or in a complex. Overall, our results unveil the partnership between Hfq and RNase R, governing the degradation and processing of rRNA molecules.

## RESULTS

### Hfq and RNase R engage in protein-protein interaction.

**FIG 1:**
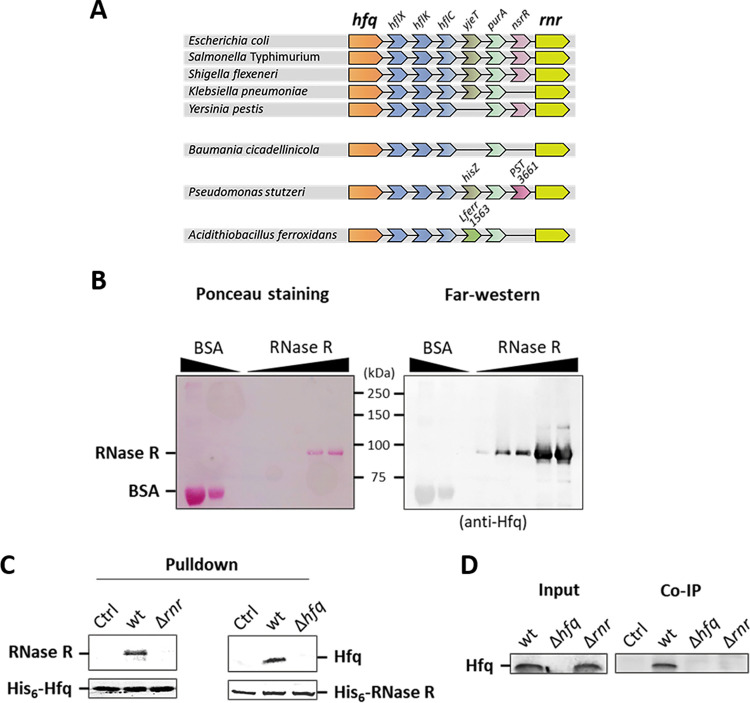
Conserved genomic proximity and protein-protein interaction of Hfq and RNase R. (A) Genomic proximity of the *hfq* and *rnr* genes in different *Gammaproteobacteria*. Genomes were analyzed with NCBI and STRING databases. *hfq*, RNA-binding protein; *hflX*, ribosome-dissociating factor; *hflK* and *hflC*, regulators of FtsH protease; *yjeT*, uncharacterized protein; *purA*, adenylosuccinate synthetase; *nsrR*, transcriptional repressor; *rnr*, RNase R. (B) Far-Western blot showing Hfq and RNase R interaction. Increasing amounts of purified RNase R (0.01, 0.05, 0.1, 0.25, and 0.5 μg) were resolved in 10% SDS-PAGE and blotted to a nitrocellulose membrane. Bovine serum albumin (BSA; 1 and 3 μg) was used as a negative control. The membrane was stained with Ponceau red (left) prior to incubation with purified Hfq in solution (45 nM final concentration) and probing with an Hfq antibody (right). Ladder information and proteins are indicated. (C) Pulldown of Hfq and RNase R. Purified His_6_-Hfq (left) or His_6_-RNase R (right) were used as the immobilized bait in Ni-NTA beads and incubated with cell lysates from wild-type (wt) or mutant strains and with binding buffer (indicated as Ctrl). Samples were analyzed by Western blotting using Hfq (54) or RNase R (13) raised antibodies. (D) Coimmunoprecipitation (Co-IP) of the Hfq/RNase R complex. A/G beads coated with anti-RNase R antibody were used to immunopurify the Hfq/RNase R complex from cell lysates of the wild-type, Δ*hfq* mutant, or Δ*rnr* mutant strain. Ctrl, negative control (no cell lysate).

A genomic analysis of Hfq (*hfq*), performed through NCBI and STRING databases, revealed that the encoding gene is located close to RNase R (*rnr*), a highly processive 3′–5′ exoribonuclease (Fig. 1A). Both genes define a conserved genomic cluster that is maintained among *Enterobacteriaceae* species as well as in other *Gammaproteobacteria*. Remarkably, the Hfq/RNase R genomic architecture is conserved even in the small genome of the obligate endosymbiont *Baumania cicadellinicola*, arguing in favor of the importance of this module for bacterial homeostasis. Genes expressing proteins with related functions that are maintained in the nearby vicinity of each other often encode interacting proteins (24). Consequently, the possibility of a protein-protein interaction between Hfq and RNase R was examined.

A far-Western approach was initially used in which increasing amounts of purified His_6_-RNase R were separated on an SDS-PAGE gel and transferred onto a nitrocellulose membrane. Following an *in situ* renaturation of the immobilized proteins, the membrane was incubated with purified His_6_-Hfq protein in solution. After extensive washing steps, the presence of Hfq bound to RNase R was probed using an anti-Hfq antiserum. A Western blot of total protein extracts shows that the anti-Hfq antiserum does not detect RNase R, arguing against a possible cross-reaction in the far-Western analysis (see Fig. S1 in the supplemental material). Hfq was clearly detected as a well-defined band that corresponds to the RNase R location (∼92 kDa) (Fig. 1B). This result indicates that both purified Hfq and RNase R can interact with each other *in vitro*.

10.1128/mBio.02398-20.1 FIG S1 Confirmation of anti-Hfq antibody specificity. Western blot analysis of total protein extracts of wild-type and single Δ*hfq* and Δ*rnr* strains is shown. Equal amounts of total protein were resolved in a 15% SDS-PAGE gel and blotted to a nitrocellulose membrane. The membrane was probed with the anti-Hfq antiserum, showing that it detects Hfq and does not react with RNase R (∼92 kDa). Ladder information and proteins are indicated. Download FIG S1, TIF file, 0.2 MB. Copyright © 2020 dos Santos et al. 2020 dos Santos et al. This content is distributed under the terms of the Creative Commons Attribution 4.0 International license.

The Hfq–RNase R interaction was then further confirmed by pulldown assays. Purified His_6_-Hfq was bound to nickel-nitrilotriacetic acid (Ni-NTA) beads and then incubated with total protein extracts. Hfq and its interaction partners were eluted with imidazole and separated on SDS-PAGE. Western blotting performed with an anti-RNase R antibody revealed that Hfq was able to interact and pull down native RNase R from the wild-type lysate (Fig. 1C, left). A reverse pulldown experiment was performed, switching the bait and prey proteins, showing that purified His_6_-RNase R also was able to pull down native Hfq from wild-type protein extracts (Fig. 1C, right). Finally, the coimmunoprecipitation approach confirmed that both proteins interact. Polyclonal antibodies raised against RNase R were immobilized on beads coated with protein A/G and subsequently used to pull down endogenous RNase R and its interacting partners from cell lysates of relevant strains. This enriched sample then was separated on SDS-PAGE, and the presence of Hfq was analyzed by Western blotting. Hfq coimmunoprecipitated with RNase R specifically from the wild-type extract, and it was absent from the control lysates of cells lacking Hfq or RNase R (Fig. 1D). Collectively, our results show that Hfq and RNase R interact with each other, forming stable complexes in cellular lysates.

### Growth defects and abnormal RNAs arise upon inactivation of Hfq and RNase R.

**FIG 2:**
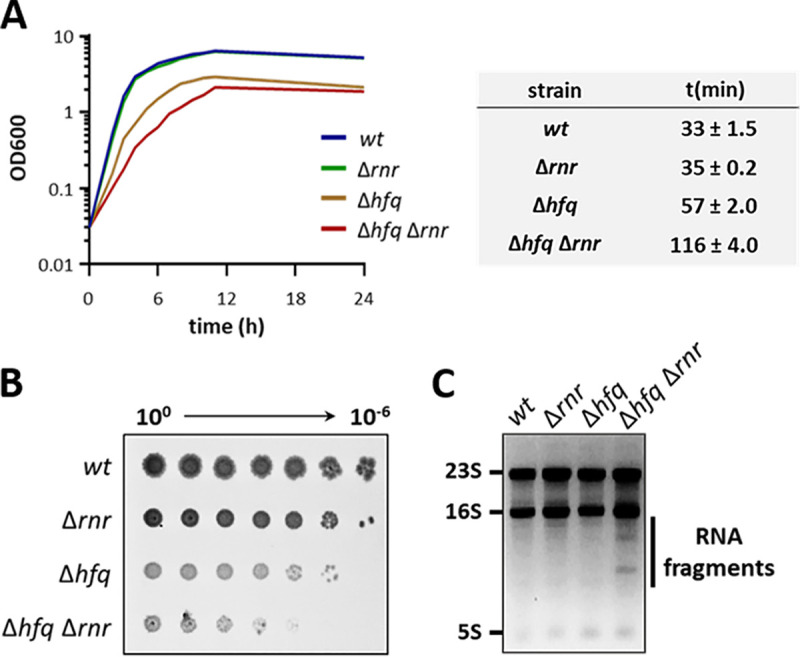
Impact of the inactivation of *hfq* and *rnr* genes in the cell. (A) Growth curve of the Δ*hfq* Δ*rnr* mutant strain compared to the parental strain (*wt*) and single mutant strains (Δ*hfq* and Δ*rnr*). Cells were grown on LB medium at 37°C. The doubling time of each strain is shown on the side. (B) Serial dilutions (with 1:10 steps) of cell cultures were spotted and grown on LB-agar plates at 37ºC. (C) Total RNA was fractionated in an agarose gel and stained with ethidium bromide. The ribosomal RNAs and accumulating RNA fragments are indicated.

Hfq and RNase R are two RNA-binding proteins with central roles in RNA biology. These proteins share at least one common substrate, the 16S rRNA (4, 23). To investigate possible functional implications of the interaction between these enzymes, a Δ*hfq* Δ*rnr* double deletion mutant was constructed and compared to the isogenic single *hfq* and *rnr* chromosomal deletions (see Fig. S2 in the supplemental material). The growth profile in liquid rich media of the Δ*hfq* Δ*rnr* strain along with its isogenic single mutants and parental strains was obtained by monitoring the optical density at 600 nm (OD_600_). As expected from previous studies, the single Δ*hfq* mutant displayed a reduced growth rate and yield (25), whereas the single Δ*rnr* mutant behaved similarly to the wild-type strain (26). Notably, the double mutant showed exacerbated growth defects with a marked increase of its doubling time, over 3 times higher than the wild type and 2 times higher than the Δ*hfq* mutant (Fig. 2A). Serial dilution plating of overnight cultures of these strains on LB-agar corroborated the growth impairment of the Δ*hfq* Δ*rnr* strain compared to the wild type and both single mutants on solid media (Fig. 2B).

10.1128/mBio.02398-20.2 FIG S2 Confirmation of the Δ*hfq* Δ*rnr* double mutant strain. (A) Deletion of *rnr* and *hfq* was confirmed by PCR using a specific pair of primers for each gene. (B) The absence of RNase Hfq and RNase R in the Δ*hfq* Δ*rnr* double mutant was additionally confirmed by Western blotting using specific anti-Hfq and anti-RNase R polyclonal antibodies. The Hfq antibody cross-reacts with a nonspecific band that migrates below Hfq in SDS-PAGE gels. Download FIG S2, TIF file, 0.3 MB. Copyright © 2020 dos Santos et al. 2020 dos Santos et al. This content is distributed under the terms of the Creative Commons Attribution 4.0 International license.

Hfq and RNase R are two important posttranscriptional regulators acting on multiple pathways of RNA metabolism (9, 21). Hence, the growth defects caused by their inactivation could arise from a disturbance in RNA metabolism. To assess this, total RNA extracts from each strain were analyzed on an ethidium bromide-stained agarose gel (Fig. 2C). The bands corresponding to the rRNA species (23S, 16S, and 5S) were identified in all strains. Strikingly, additional bands migrating below the 16S rRNA were specifically present in the Δ*hfq* Δ*rnr* but not in the wild-type or single mutant strains. These RNA molecules must accumulate to high levels to be clearly visible under UV light in an ethidium bromide-stained gel not requiring the use of specific probes. This set of results points toward a functional relationship for the conservation of the Hfq/RNase R genomic cluster, since the disruption of both genes leads to marked growth defects and abnormal accumulation of RNA species.

### Hfq and RNase R cooperate in the elimination of rRNA-derived fragments.

**FIG 3:**
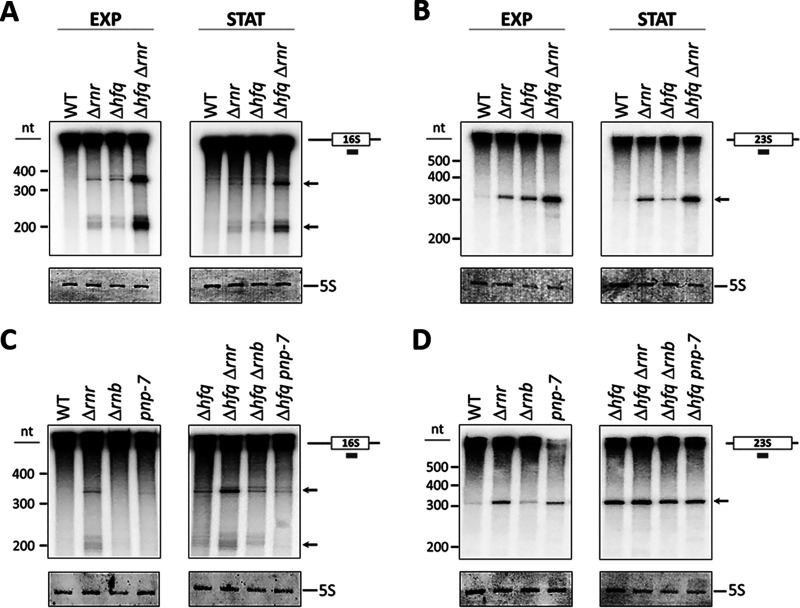
Hfq and RNase R cooperate in the degradation of 16S and 23S rRNA fragments. (A and B) RNA extracted from both exponential (EXP)- and stationary (STAT)-phase cultures was analyzed by Northern blotting on 8% polyacrylamide–7 M urea gels. rRNA fragments were detected through the use of specific 16S (A) or 23S (B) probes that hybridize to the central region of each rRNA molecule, depicted on the right. (C and D) Analysis of rRNA pattern in total RNA extracted from exoribonuclease mutants expressing or not expressing *hfq*. Specific oligonucleotide probes were used to detect 16S (C) and 23S (D) rRNA fragments. Fragments are indicated with arrows. Two specific 16S-derived fragments (∼350 and ∼200 nt) and one 23S-derived fragment (∼300 nt) accumulate to high levels in the Δ*hfq* Δ*rnr* mutant. Ladder information is depicted on the left, and methylene blue staining of the same blotted membrane showing the 5S rRNA is presented below each panel.

The accumulation of RNA species observed in the Δ*hfq* Δ*rnr* double mutant could derive from rRNA, the most abundant class of cellular RNAs. To test this, Northern blot analysis was performed using radioactive oligonucleotide probes complementary to the 16S, 23S, and 5S rRNAs. Although rRNA levels are higher during exponential growth, it is known that the levels of Hfq and RNase R are increased in stationary phase, with both proteins acting as key regulators of this growth stage (26, 27). Hence, both exponential- and stationary-phase cultures were analyzed in the forthcoming experiments. Total RNA from exponential- and stationary-phase cells was fractionated on denaturing polyacrylamide gels and transferred onto a nitrocellulose membrane to subsequently be probed for each of the three rRNAs (Fig. 3). In addition to the full-length mature rRNAs and other large processing or degradation fragments that comigrate in the gel, lower-molecular-weight RNA molecules were also detected in the single and double mutants when using the 16S and 23S rRNA probes (Fig. 3A and B). Two different rRNA fragments, with approximately 200 and 350 nucleotides (nt) each, were identified when using the 16S probe (Fig. 3A), whereas one rRNA fragment of approximately 300 nt was detected in the 23S rRNA analysis (Fig. 3B). These 16S and 23S internal fragments are degradation products that arise in the single and double mutant strains and are not detected in the wild type. However, they accumulate to much higher levels in the double Δ*hfq* Δ*rnr* strain in both exponential- and stationary-phase cells, showing that the action of Hfq and RNase R on rRNA removal is active on both growth phases. In contrast, no differences were found when the 5S rRNA was studied (data not shown).

RNase R is one of three major 3′–5′ exoribonucleases in E. coli, along with RNase II and PNPase (9). To study the possible involvement of these exoribonucleases in this novel rRNA degradation pathway, we next examined if inactivation of RNase II (*rnb*) or PNPase (*pnp*) alone or together with *hfq* deletion would affect the accumulation pattern of the rRNA fragments. In contrast to what was shown previously for RNase R deletion in cells with Hfq, inactivation of RNase II or PNPase did not result in the accumulation of degradation intermediates. This was also the case upon simultaneous inactivation of Hfq (Fig. 3A and C). Some accumulation of the 23S-derived fragment was observed in single RNase II or PNPase mutants but not to the high level resulting from the inactivation of RNase R (Fig. 3B and D). Collectively, our findings suggest that the major 3′–5′ exoribonuclease responsible for the degradation of the intermediates that accumulate upon inactivation of Hfq is RNase R (and not RNase II or PNPase).

### Processing of rRNA precursors is affected by the double inactivation of Hfq and RNase R.

**FIG 4:**
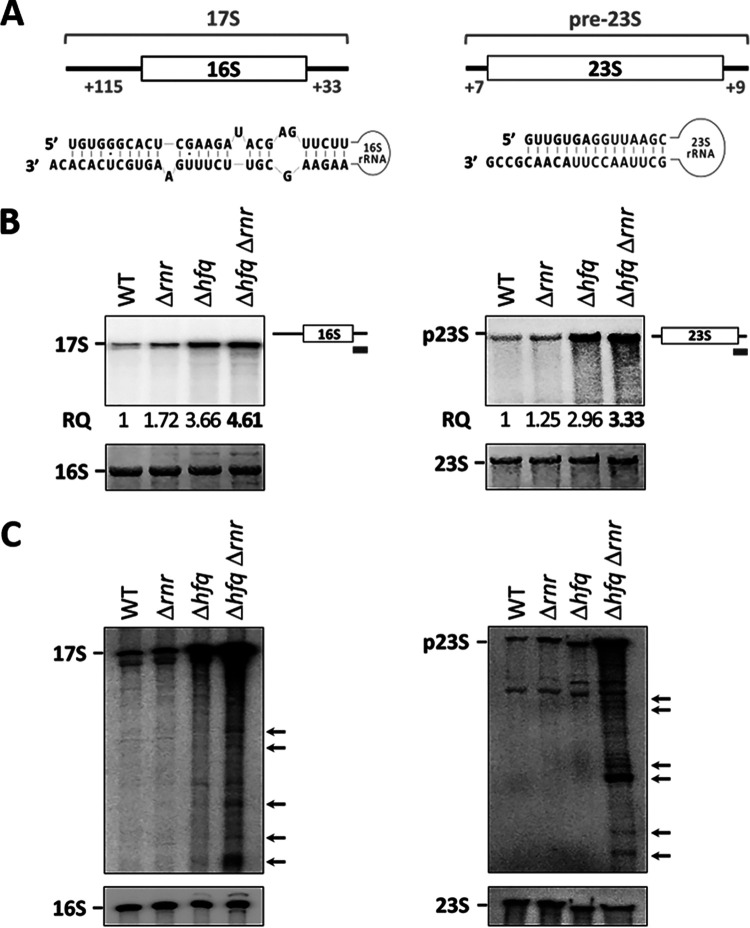
17S and pre-23S precursors accumulate in the Δ*hfq* Δ*rnr* mutant. (A) Extra nucleotides present in the 17S and pre-23S precursor rRNAs. A scheme of the hybridization between the extra nucleotides of the 5′ and 3′ ends of each rRNA precursor is presented below. (B) Four percent polyacrylamide–7 M urea gel and Northern hybridization analysis of the 17S rRNA and pre-23S rRNA precursors (left and right, respectively) extracted from stationary-phase cultures. Specific probes directed against the extra nucleotides in the 3′-end rRNA precursor were used in Northern blot hybridizations, as depicted on the right. Relative quantification (RQ) of three independent experiments for precursor rRNA accumulation is shown below, as well as methylene blue staining of the same blotted membrane showing the mature 16S and 23S rRNAs. (C) Northern blot analysis of rRNA precursor fragments. Specific 3′-end probes for the 17S (left) or pre-23S (right) rRNAs were used. Fragments that accumulate specifically in the double mutant are indicated by arrows on the side.

Previous work established a role for either Hfq or RNase R in rRNA processing, a critical step in the production of mature functional rRNA molecules (28). The 16S precursor, termed 17S rRNA, has 115 extra nucleotides at the 5′ end and 33 extra nucleotides at the 3′ end that need to be trimmed to give rise to the mature 16S rRNA. Similarly, the pre-23S precursor has 7 nt and 9 nt extra at the 5′ and 3′ ends, respectively, that are also eliminated during 23S maturation. In both cases, the sequences that are not yet processed at one end can base pair with the other at the opposite end (Fig. 4A). This secondary structure is believed to protect against some ribonucleases obstructing the correct processing steps (28). Northern blot analysis of total RNA extracts was used to assess the levels of these rRNA precursors in cells lacking Hfq, RNase R, or both. Specific oligonucleotide probes that hybridize to the 3′ trailer sequence of each rRNA precursor were used (Fig. 4B). Because of a shorter 3′ trailer, our pre-23S rRNA probe necessarily carried a partial complementarity to the 3′ end of the 23S sequence. Nevertheless, we found that this probe hybridizes much more strongly with the pre-23S rRNA than with the mature 23S molecule. The 17S rRNA levels did not change in the Δ*rnr* single mutant compared to the wild type. On the other hand, inactivation of Hfq led to the accumulation of this rRNA precursor. However, the combined inactivation of Hfq and RNase R led to even higher levels of the 17S rRNA, as observed in the Δ*hfq* Δ*rnr* mutant. Strikingly, similar results were obtained when analyzing the maturation of the 23S rRNA. Additionally, we used a probe complementary to the 5′ end leader of the 17S rRNA to assess if the accumulation observed corresponded to the full-length precursor (Fig. S3). Indeed, the full-length 17S rRNA was found to accumulate to high levels in the double mutant. Altogether, these results indicate that Hfq and RNase R are also involved in the processing of the 16S and 23S rRNAs.

10.1128/mBio.02398-20.3 FIG S3 Accumulation of 17S rRNA in the Δ*hfq* Δ*rnr* mutant using a 5′ specific probe. Four percent polyacrylamide–7 M urea gel and Northern hybridization analysis of the 17S rRNA precursors extracted from stationary-phase cultures are shown. A specific probe directed against the 5′-end rRNA of the 17S rRNA precursor was used in Northern blot hybridizations, as depicted on the right. Download FIG S3, EPS file, 1.1 MB. Copyright © 2020 dos Santos et al. 2020 dos Santos et al. This content is distributed under the terms of the Creative Commons Attribution 4.0 International license.

An overexposure of membranes hybridized with the probes directed against the 3′ end of either the 17S rRNA or pre-23S rRNA revealed that several shorter rRNA species carrying the 3′ end precursor sequence accumulated specifically on the Δ*hfq* Δ*rnr* mutant but not on the wild-type or single mutants (Fig. 4C). Therefore, Hfq and RNase R are further required for the degradation of rRNA fragments that retain the 3′ end of precursor sequences. Collectively, these results not only demonstrate for the first time the cooperation of Hfq and RNase R in the processing of rRNA but also show that their inactivation results in the accumulation of rRNA fragments and rRNA maturation defects.

### Hfq and RNase R are required for correct 70S ribosome assembly.

**FIG 5:**
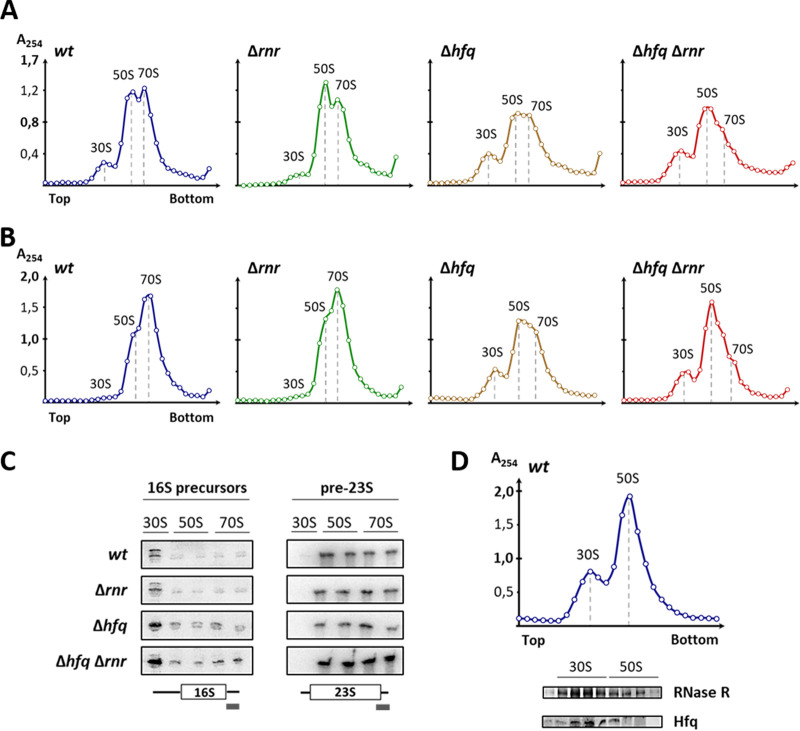
Ribosome assembly defects arise upon double inactivation of Hfq and RNase R. (A and B) Fifteen to 50% sucrose density gradient analysis of ribosomal particles extracted from exponential-phase (A) and stationary-phase (B) cells analyzed under associative conditions (10 mM Mg^2+^) to promote subunit association into 70S ribosomes. Gradient fractions were collected, and absorbance at 254 nm was measured and plotted. Top and bottom denote the lower and higher sucrose concentrations in the gradient, respectively. Gradient analyses shown are representative of at least three independent experiments. (C) RNA was isolated from representative gradient fractions of each ribosomal particle (30S, 50S, and 70S), and Northern blot analysis was performed to assess the presence of 17S and pre-23S precursor rRNAs (left and right, respectively). Each set of wild-type and single and double mutant strains belongs to the same blotted membrane using a specific probe for the 3′ end extension of either the 17S or pre23S rRNA, as depicted below. (D) Ten to 30% sucrose density gradient of wild-type ribosomal particles extracted from stationary-phase cells, analyzed under dissociative conditions (0.1 mM Mg^2+^), to promote the complete dissociation of 70S ribosomes into the free 30S and 50S subunits. The protein content of representative gradient fractions was isolated and probed by Western blotting for the presence of RNase R and Hfq using specific antibodies. Ribosomal particles are indicated on top of the analyzed fractions and of each peak.

The accumulation of rRNA precursors is a hallmark of ribosome biogenesis defects (29). We next analyzed if the rRNA processing defects detected in the Δ*hfq* Δ*rnr* strain would correlate with changes in the ribosome profiles. Ribosomes isolated from both exponential- and stationary-phase cultures of the wild-type and mutant strains were analyzed and compared using sucrose gradient ultracentrifugation (Fig. 5A and B). Under conditions that favor ribosome association (10 mM Mg^2+^), most ribosome subunits are associated with 70S particles in the wild type. A similar profile was obtained for the single RNase R mutant. In contrast, the Hfq mutant revealed a reduction in the amount of 70S ribosomes, as we have previously reported (23). Notably, the combined inactivation of Hfq and RNase R resulted in the increase of the 30S subunit peak as well as in an elevation of the 50S subunit peak, accompanied by a greater reduction of the 70S particles. Indeed, the higher peak in the Δ*hfq* Δ*rnr* strain actually corresponds to the 50S subunit population rather than to 70S ribosomes, as confirmed by the analysis of the RNA in the gradient fractions (Fig. S4). These results indicate, for the first time, that RNase R interacts functionally with Hfq in ribosome biogenesis, regardless of the growth stage.

10.1128/mBio.02398-20.4 FIG S4 Identification of peaks corresponding to ribosomal particles in sucrose gradients. Ribosomes were analyzed by ultracentrifugation of 15 to 50% sucrose density gradients under associative conditions (10 mM Mg^2+^). Gradient fractions were collected, and absorbance at 254 nm was measured and plotted. The RNA content of representative fractions for each ribosomal particle (30S, 50S, and 70S) was isolated and analyzed on a 1.5% agarose gel stained with ethidium bromide. The rRNA species are shown below each gradient and confirm the identity of the peaks along the gradient. Download FIG S4, TIF file, 0.6 MB. Copyright © 2020 dos Santos et al. 2020 dos Santos et al. This content is distributed under the terms of the Creative Commons Attribution 4.0 International license.

To assess if incompletely processed molecules were being incorporated, ribosomes of each strain were separated on sucrose gradients, and representative fractions from each peak (30S, 50S, and 70S) were collected. The RNA content from each fraction was extracted and analyzed by Northern blotting using specific probes (Fig. 5C). Unlike total RNA extracts, these samples allow for an assessment of the maturation status of the rRNAs incorporated into ribosomal particles (30, 31). Hence, we could detect two 16S precursor forms with unprocessed 3′ ends, with the upper band corresponding to the 17S rRNA. Although precursor 17S rRNA was detected in free 30S subunits of the wild-type and Δ*rnr* strains, it was present at higher levels in the Δ*hfq* mutant. Notably, this accumulation was greater in the case of the Δ*hfq* Δ*rnr* double mutant, in which 17S rRNA misincorporation extended into 70S particles. Similarly, pre-23S precursor rRNA was detected not only in 50S free subunits but also in 70S particles of all strains, but particles isolated from the double mutant showed higher levels pre-23S rRNA incorporation. Nevertheless, we could not exclude the existence of cross-contamination between nearby fractions due to the collection method applied. Hence, the detection of 17S rRNA in 50S subunit particles most likely is due to the incomplete separation of the ribosomal particle peaks (Fig. S4). However, as 30S and 70S peaks are very well separated, detection of 17S rRNA in the latter reflects the incorporation of this precursor in 70S ribosomes.

To evaluate an interaction between Hfq and RNase R with the ribosomes, the sedimentation profile of each protein isolated from ribosomal fractions was analyzed. Individual fractions from the wild-type strain ribosome profile were recovered under conditions that promote subunit dissociation (0.1 mM Mg^2+^). The localization of Hfq and RNase R across the gradient was analyzed by specific antibodies (Fig. 5D). Data show that both Hfq and RNase R predominantly copurify with the 30S subunit and, to a lesser extent, comigrate with the 50S subunit. However, most probably the detection of Hfq in 50S fractions can be explained by the presence of contaminant 30S particles from nearby fractions. Overall, this result suggests that both proteins can be present in a complex associated with ribosomal particles, namely, the 30S subunits. Consequently, the interaction between Hfq-RNase R-30S subunit is suggested to be important for the processing of 17S and pre-23S precursor rRNAs. Perturbation of this interaction leads to the accumulation of precursor rRNAs in ribosome subunits, which may prevent the assembly of the 70S ribosomes or could cause subunit dissociation, resulting in lower levels of mature ribosomes.

## DISCUSSION

**FIG 6:**
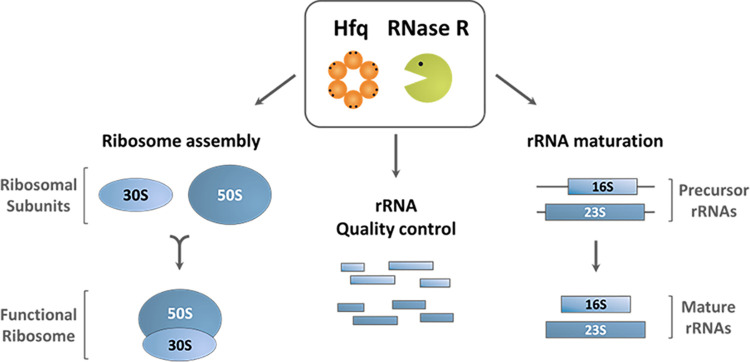
Model for the roles of Hfq and RNase R cooperation in RNA quality control. Hfq and RNase R are required for stable RNA quality control and degradation. Both enzymes are required for the normal turnover of tRNA molecules and are key factors in a novel rRNA quality control mechanism that eliminates abnormal rRNA fragments from the cell. Notably, no other major exoribonuclease seems to be involved in this rRNA degradation pathway. Upon Hfq and RNase R inactivation, unprocessed 17S and pre-23S rRNAs highly accumulate in the cell, and the levels of intact 70S ribosomes are strongly reduced. This correlates with a marked increase in the population of free 30S and 50S subunits that are unable to associate. These findings strongly suggest the existence of severe defects in the ribosome biogenesis process in the absence of Hfq and RNase R.

In this work, we have shown that the RNA-binding proteins Hfq and RNase R participate in rRNA quality control, governing previously unrecognized processing and degradation pathways (Fig. 6). RNase R is a highly processive 3′–5′ exoribonuclease unique for its ability to degrade structured RNAs (3, 16, 26). Hfq is an RNA chaperone involved in small noncoding RNA biology and was recently shown to be an important factor in rRNA processing (21–23, 32, 33). Our results now show that Hfq and RNase R are partners in a previously unrecognized surveillance mechanism that eliminates detrimental rRNA fragments. Efficient processing and removal of decay intermediates is a crucial step in rRNA maturation, as their incorporation into ribosomes could lead to their malfunction and perturbation of cell growth (3, 12). Single deletion mutants of Hfq or RNase R accumulate both 16S- and 23S-derived fragments; however, this accumulation is markedly higher in the Δ*hfq* Δ*rnr* mutant (Fig. 3). Therefore, these RNA-binding proteins are involved in coordinated rRNA degradation pathways. This correlates with the extensive growth defects of the Δ*hfq* Δ*rnr* mutant in both liquid and solid rich media (Fig. 2), since the accumulation of such rRNA fragments is often toxic to the cell (3, 12). Strikingly, inactivation of RNase II or PNPase had little effect on the accumulation of rRNA species compared to RNase R depletion, regardless of the expression of Hfq.

In a similar manner, Hfq and RNase R are found to affect the maturation of rRNA precursors. Even though the inactivation of Hfq or RNase R alone leads to an accumulation of rRNA precursors, the combined inactivation of Hfq and RNase R causes an even larger accumulation of both the 17S rRNA and the pre-23S rRNA (Fig. 4). The absence of Hfq and RNase R could not be compensated by any of the remaining rRNA maturation factors in the cell, similar to what was observed with the degradation of rRNA fragments. Remarkably, the full-length 17S precursor is the major 16S precursor form accumulating in total RNA extracts of the double mutant (Fig. 4B; see also Fig. S3 in the supplemental material). This agrees with the view that processing at the 3′ end of the 17S rRNA favors subsequent 5′-end processing (34, 35). However, 5′-end processing may still occur independently of the 3′ maturation status (31). Novel rRNA maturation pathways are still being uncovered (35–37), and the recent implication of the 5′–3′ exoribonuclease, RNase AM, in the 5′-end processing of both 16S and 23S rRNAs may offer an alternative route for precursor maturation (38).

rRNA is the main component of ribosomes, and the processing of precursor rRNA molecules is intricately interconnected with ribosome biogenesis. Defects in ribosome assembly are often associated with misprocessing of rRNA (29). Indeed, inactivation of Hfq and RNase R not only results in the strong accumulation of rRNA precursors but also causes a sharp reduction in the levels of 70S ribosomes (Fig. 5). Even though the population of free 30S and 50S subunits increases in the double Δ*hfq* Δ*rnr* mutant, these subunits do not associate into 70S particles. Our findings point to the existence of severe assembly defects in the Δ*hfq* Δ*rnr* double mutant strain that prevent subunit association into active 70S ribosomes, regardless of the growth phase analyzed. However, such defects are more notorious in stationary phase than in exponential phase (Fig. 5A and B). Hfq and RNase R are known regulators of the stationary phase (26, 39, 40), suggesting that both proteins have more important functions at this late growth stage. Moreover, the ribosome association defects are also consistent with our observation that both the 17S and pre-23S rRNA precursors are highly enriched in ribosomal fractions isolated from the double Δ*hfq* Δ*rnr* mutant compared to the wild type or any of the single mutants (Fig. 5C). Although we could detect two 16S precursor forms with unprocessed 3′ ends but either with processed or unprocessed 5′ ends, only the full-length unprocessed 17S rRNA accumulates to higher levels in 30S subunits and 70S ribosomes upon inactivation of Hfq and RNase R.

Although RNase R is highly efficient in the degradation of most RNAs, its processive exoribonuclease activity depends on the presence of a 3′-end overhang on the RNA substrate (41). However, rRNA fragments usually result from endonucleolytic cleavages and retain extensive secondary structures, which may hinder access to the 3′ end (4). Therefore, it is plausible that the RNA chaperone Hfq acts to remodel the highly structured rRNA molecules, providing them substrates that become suitable targets for RNase R. Furthermore, RNA secondary structures also play important roles in the maturation of rRNA precursors. It has been described that the flanking sequences found on both ends of precursor rRNAs have the ability to form intramolecular interactions that sequester the 3′ end in a double-stranded region (28). Cooperation between Hfq and RNase R may help overcome RNA secondary structures and make the 3′ end available for remodeling, promoting the correct processing of rRNA molecules. Notably, the binding and action of various 30S-associated ribosome biogenesis factors was also described to impact the maturation of the 16S rRNA (29, 42). These include the Era GTPase, which was previously shown to bind to the 3′-end trailer of the 17S rRNA and protect it from premature degradation (36, 37). One can envision Hfq and RNase R competing with Era for accessibility to the 3′-end trailer to initiate the rRNA processing step. The absence of both Hfq and RNase R then would result in the accumulation of 16S precursors with unprocessed 3′ ends. Because redundant maturation pathways are part of rRNA maturation, a fraction of these molecules would still undergo alternative processing mediated by other RNases (18, 28, 31, 36).

Interestingly, Hfq seems to act as a hub for interacting protein partners related to the RNA machinery (22). For example, Hfq association with the endonuclease RNase E was proposed to be important for sRNA-mediated regulation (43). Hfq was also found to bind *in vitro* and regulate the biosynthetic activity of the poly(A) polymerase I (44). We now show that Hfq associates with RNase R in a novel protein complex, which is detected both *in vitro* and in cell lysates (Fig. 1). This is in line with a recent report that identified protein-protein interactions with E. coli Hfq, in which the Hfq/RNase R complex was also detected and suggested to require an RNA third partner (45). We also show that Hfq and RNase R cosediment with 30S subunits, which suggests that an Hfq/RNase R/30S subunit complex is formed. In this case, either the 16S rRNA acts as a third partner for the Hfq/RNase R complex or Hfq and RNase R could be interacting with the 30S subunit through the ribosomal protein S12 (19). Although our findings suggest that such a complex plays a role in the metabolism of stable RNA, we recognize that this is not the only active pathway. In fact, Hfq and RNase R can act in concurrent pathways working in an additive manner, as the double inactivation of both activities results in a stronger phenotype than depletion of only one.

A surveillance mechanism mediated by Hfq and RNase R that is active in the maturation of rRNA precursors is highly advantageous for the cell, as it acts as a primary quality control pathway on ribosomal subunit production, preventing translational errors and consequent superfluous energetic costs. Could Hfq and RNase R play a broader role in the metabolism of other stable RNA molecules? Our preliminary results showed that Hfq and RNase R are involved in controlling the levels of some tRNAs, although we could not find any defects in tRNA processing (Fig. S5). A recent report showed that the accumulation of unprocessed tRNA precursors triggers the stringent response along with a drop in GTP levels, which in turn affect GTPases acting on 30S maturation and lead to the accumulation of 16S rRNA precursors (46). However, as only increased levels of mature tRNAs and not their precursor forms are found in the Δ*hfq* Δ*rnr* double mutant, our results suggest that Hfq and RNase R act directly on rRNA processing and ribosome assembly rather than indirectly as a consequence of tRNA processing defects.

10.1128/mBio.02398-20.5 FIG S5 Accumulation of various tRNA species in the Δ*hfq* Δ*rnr* double mutant. Total RNA extracted from exponential-phase cultures was analyzed by Northern blotting in an 8% polyacrylamide–7 M urea gel using oligonucleotide probes specific for each tRNA species indicated on the top of each panel. Below each panel is the relative quantification (RQ) of three independent experiments for assessing tRNA levels as well as methylene blue staining of the same blotted membrane showing the 5S rRNA. Download FIG S5, TIF file, 0.4 MB. Copyright © 2020 dos Santos et al. 2020 dos Santos et al. This content is distributed under the terms of the Creative Commons Attribution 4.0 International license.

Overall, these results indicate, for the first time, that RNase R functionally interacts with Hfq in ribosome biogenesis, regardless of the growth stage. Our data suggest that Hfq and RNase R are involved in multiple facets of stable RNA metabolism and can also act as a broader surveillance mechanism for the degradation of structured RNAs. Disruption of both Hfq and RNase R leads to exacerbated defects in rRNA maturation or rRNA and tRNA degradation compared to the defects of any of the single mutants, which indicates the participation of these enzymes in reciprocal RNA degradation pathways. Thus, the high conservation of the RNA-binding proteins Hfq and RNase R suggests a wider involvement for these proteins in stable RNA metabolism throughout evolution and may offer a previously unrecognized pathway to regulate highly structured RNAs.

## MATERIALS AND METHODS

### Bacterial strains and growth.

All experiments use derivatives of E. coli K-12 strain MG1693 (47). Variants lacking Hfq and/or exoribonucleases were constructed using the λ-Red recombination system (48) and P_1_ transduction (49). Two alleles of *hfq* were used throughout this study (23, 47). Details on the construction of the Δ*hfq* Δ*rnr* double mutant are given in the supplemental material along with the list of primers (see Table S1).

10.1128/mBio.02398-20.6 TABLE S1 Primers used in this work. Download Table S1, XLSX file, 0.01 MB. Copyright © 2020 dos Santos et al. 2020 dos Santos et al. This content is distributed under the terms of the Creative Commons Attribution 4.0 International license.

### Growth.

Bacteria were grown at 37ºC in Luria-Bertani (LB) medium supplemented with thymine (50 μg/ml). Antibiotics were present at the following concentrations when needed: 25 μg/ml chloramphenicol, 25 μg/ml kanamycin, 10 μg/ml tetracycline, and 100 μg/ml ampicillin. In the dilution plating assays, serial dilutions were made in 10-fold increments and immediately spotted onto LB-agar plates using a replica plater. The plates were incubated at 37ºC for ∼36 h. Further details are provided in the supplemental material.

### RNA extraction and Northern hybridization analysis.

Total RNA was prepared from E. coli cells by the phenol-chloroform method, and Northern blot analysis was carried out as previously described (50). For rRNA fragment and tRNA analyses, 1 to 3 μg of total RNA extracts were resolved 8% polyacrylamide–7 M urea gels ran in Tris-borate-EDTA (TBE) buffer; 4% polyacrylamide–7 M urea gels in TBE buffer were used as previously described for the identification of rRNAs precursors (42). In Northern blots, all lanes being compared in one panel correspond to the same gel, but the lane order may differ from the original loading for presentation purposes. DNA oligonucleotide probes were labeled with [γ-^32^P]ATP (Perkin-Elmer) at the 5′ end using T4 polynucleotide kinase (Fermentas). Membranes were hybridized at 42°C and analyzed with a PhosphorImager (Fujifilm). Oligonucleotide probes used in this work are listed in Table S2.

10.1128/mBio.02398-20.7 TABLE S2 Oligonucleotide probes used in this work. Download Table S2, XLSX file, 0.02 MB. Copyright © 2020 dos Santos et al. 2020 dos Santos et al. This content is distributed under the terms of the Creative Commons Attribution 4.0 International license.

### Ribosome extraction and ribosome profile analysis.

The ribosome isolation procedure was adapted from reference 51. Purified ribosomes were analyzed in 15% to 50% (wt/vol) sucrose gradients with 10 mM MgCl_2_ (associative conditions) or in 10% to 30% (wt/vol) sucrose gradients with 0.1 mM MgCl_2_ (dissociative conditions). Samples were centrifuged in a Beckman ultracentrifuge SW28 rotor for 16 h at 24,000 rpm at 4°C. Fractions (1 ml) were collected from the top and quantified by *A*_254_ measurement on a NanoDrop machine. A detailed description is provided in the supplemental material.

### Protein purification.

His-tagged RNase R and His-tagged Hfq were purified on Ni-NTA columns (GE Healthcare) by following the protocols previously described (11, 52). Details are presented in the supplemental material.

### Pulldown assay.

Purified His_6_-RNase R or His_6_-Hfq was bound to Ni-NTA beads, and relevant cell lysates were incubated overnight at 4°C with gentle rocking. Bound proteins were then washed, eluted, and analyzed by Western blotting and probed with Hfq or RNase R antibodies. Further details are provided in the supplemental material.

### Coimmunoprecipitation of RNase R and Hfq.

Cell lysates were incubated with RNase R antibody bound to protein A/G agarose beads based on the instructions of the Pierce Crosslink immunoprecipitation kit (Thermo Scientific). Eluted Hfq in complex with RNase R was identified by Western blotting. Details are provided in the supplemental material.

### Far-Western blot analysis.

RNase R/Hfq protein interaction was assessed by a far-Western blotting technique as previously described (53). Purified RNase R and Hfq were used as prey and bait proteins, respectively. Hfq bound to renatured RNase R on nitrocellulose membranes was detected by Western blotting with Hfq antibodies. Details are provided in the supplemental material.

10.1128/mBio.02398-20.8 TEXT S1 Supplemental experimental procedures. Download Text S1, DOCX file, 0.01 MB. Copyright © 2020 dos Santos et al. 2020 dos Santos et al. This content is distributed under the terms of the Creative Commons Attribution 4.0 International license.
