# Supplementary figures and images for: Hfq and RNase R Mediate rRNA Processing and Degradation in a Novel RNA Quality Control Process
Source: mBio. 2020 Oct 20;11(5):e02398-20. doi: 10.1128/mBio.02398-20 (PMC7587437; doi:10.1128/mBio.02398-20)

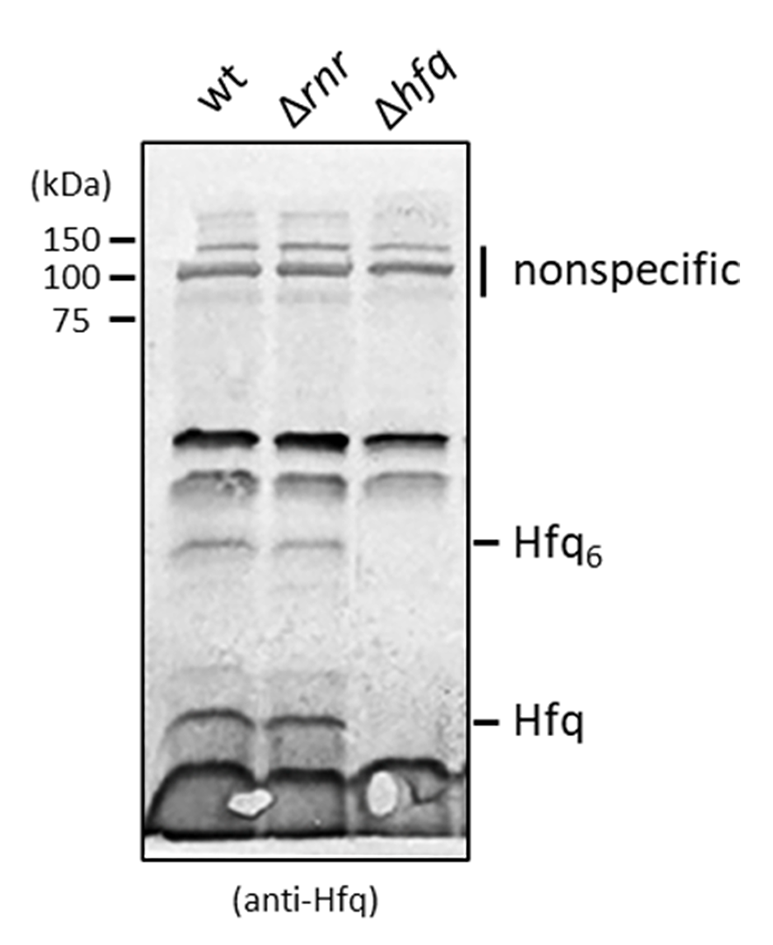

Supplement: FIG S1 [file mBio.02398-20-sf001.tif]

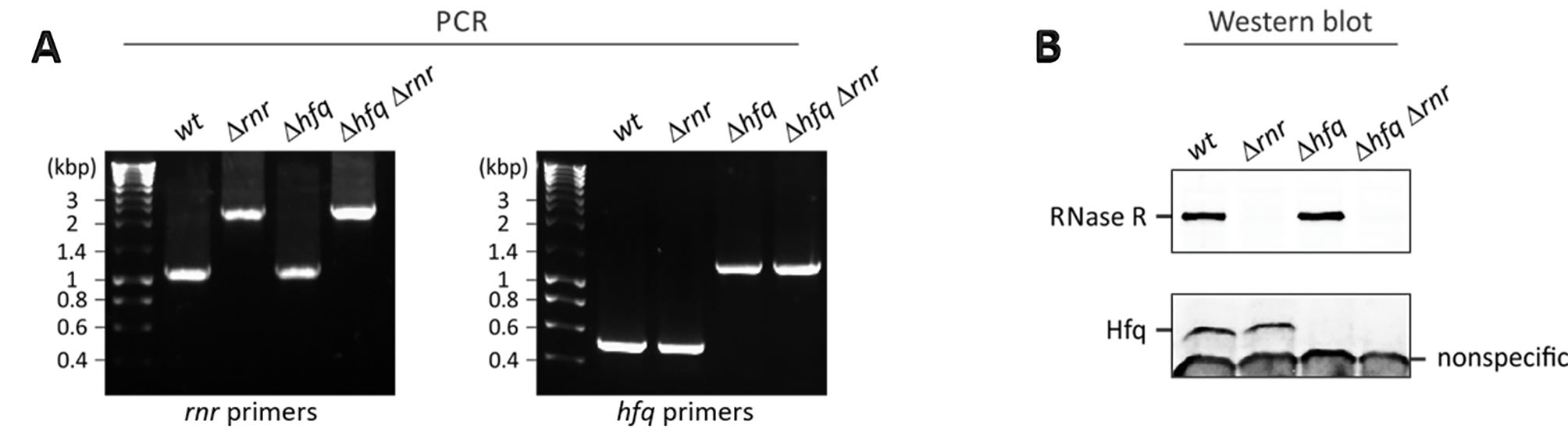

Supplement: FIG S2 [file mBio.02398-20-sf002.tif]

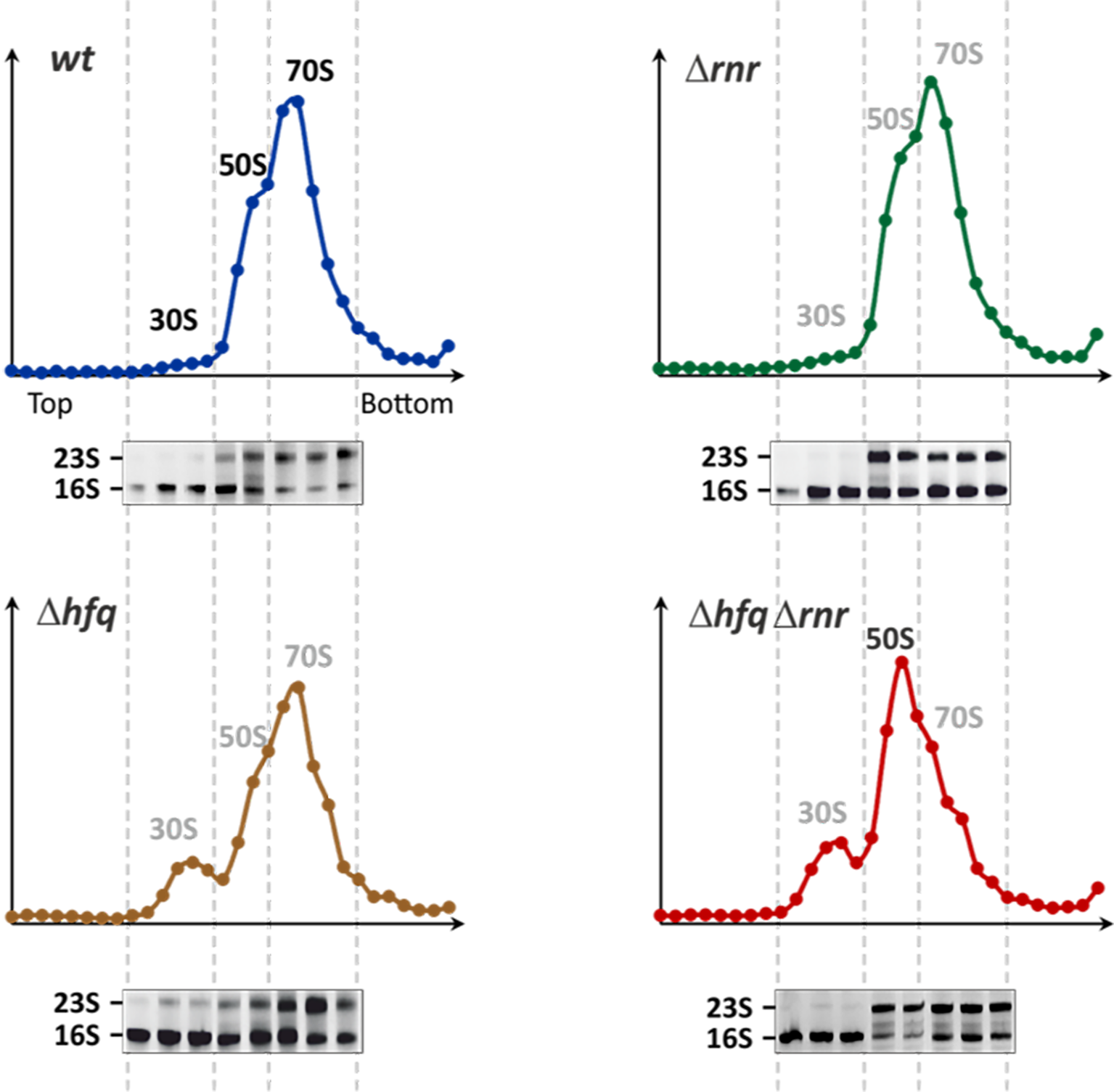

Supplement: FIG S4 [file mBio.02398-20-sf004.tif]

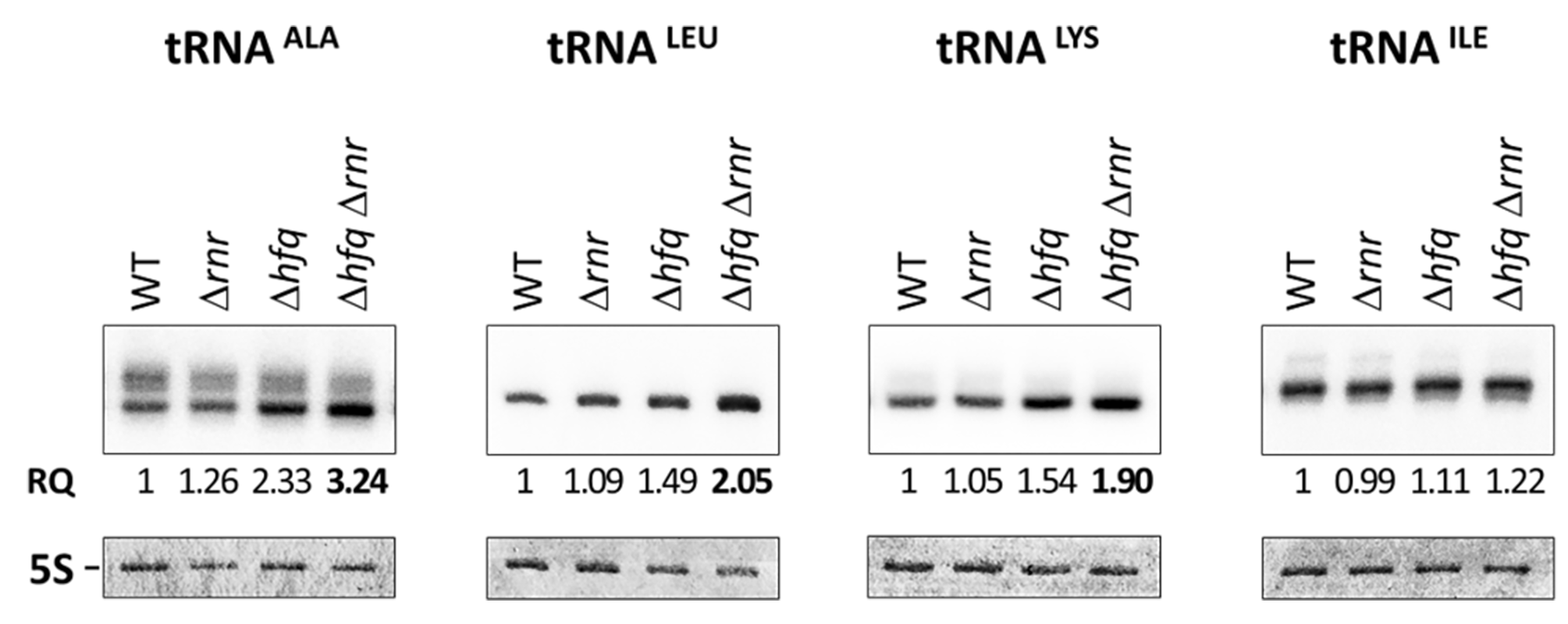

Supplement: FIG S5 [file mBio.02398-20-sf005.tif]
