# Supplementary material for: Hfq and RNase R Mediate rRNA Processing and Degradation in a Novel RNA Quality Control Process
Source: mBio. 2020 Oct 20;11(5):e02398-20. doi: 10.1128/mBio.02398-20 (PMC7587437; doi:10.1128/mBio.02398-20)
Supplement: TEXT S1 [file mBio.02398-20-s0001.docx]

**Supplemental Material**

**Hfq and RNase R mediate rRNA processing and degradation in a novel RNA quality control process**

Ricardo F. dos Santos^1^, José M. Andrade^1,#^, Joana Pissarra^1,*^, Murray P. Deutscher^2^

and Cecília M. Arraiano^1,#^

^1^ Instituto de Tecnologia Química e Biológica António Xavier, Universidade Nova de Lisboa, Avenida da República, 2780-157 Oeiras, Portugal

^2^ Department of Biochemistry and Molecular Biology, Miller School of Medicine, University of Miami, Miami, FL 33101, USA

* Present address: Institut de Recherche Pour Le Développement (IRD), UMR INTERTRYP IRD-CIRAD, University of Montpellier, F-34398 Montpellier, France

RFdS and JMA contributed equally to this work

**Running head**: Stable RNA metabolism driven by Hfq and RNase R

# Address correspondence to Cecília M. Arraiano, [cecilia@itqb.unl.pt](mailto:cecilia@itqb.unl.pt); Tel: (+351) 21 446 95 47

# Address correspondence to José M. Andrade, [andrade@itqb.unl.pt](mailto:andrade@itqb.unl.pt); Tel: (+351) 21 446 95 48

**Keywords**: Hfq/ RNase R/ RNA quality control/ ribosomal RNA/ RNA processing/ RNA maturation

**Supplemental Experimental Procedures**

**Bacterial strains**

All experiments use derivatives of *E. coli* K-12 strain MG1693 (3). Deletion mutant strains were confirmed by PCR and Western blotting (Fig. S1). P1-mediated transduction was used to transfer mutations to a fresh *E. coli* wild-type background as well as to construct multiple mutants. Primers used in this work are provided in Table S1.

**Growth**

Bacteria were grown at 37ºC in Luria-Bertani (LB) medium supplemented with thymine (50 µg/ml). Antibiotics were present at the following concentrations when needed: 25 µg/ml for chloramphenicol, 25 µg/ml for kanamycin, 10 µg/ml for tetracycline and 100 µg/ml for ampicillin. Fresh LB media was inoculated with overnight cultures of isolated fresh grown colonies to an initial OD_600_ ~ 0.03. Cells were incubated with orbital shaking and grown to an optical density of 0.4 (exponential phase cells) or until 10 hours after reaching an OD_600_ of 0.1 (stationary phase). In the growth curves, cell doubling times were calculated as DT = (t2-t1) x [log2/ (log OD_600_(t2) – logOD_600_(t1)], using the period of exponential growth. In the dilution plating assays, serial dilutions were made in 10-fold increments and immediately spotted onto LB-agar plates using a replica plater. The plates were incubated at 37ºC for ~36h.

**Ribosome Extraction**

Ribosome isolation was adapted from Zundel et al., 2009 (4). Cell pellets were resuspended in ice-cold buffer A (50 mM Tris-Cl at pH 7.5, 10 mM MgCl_2_, 100 mM NH_4_Cl, 0.5 mM EDTA, and 6 mM 2-mercaptoethanol) with the addition of Complete Mini Protease Inhibitor Cocktail EDTA-free (Roche) and lysed by four passes in a French Press. TurboDNase (Ambion) was added to the lysate. The cell lysate was centrifuged twice at 14,000 rpm for 10 min at 4ºC. The clarified lysate was layered over a 36% sucrose cushion composed of buffer B (50 mM Tris-Cl at pH 7.5, 10 mM MgCl_2_, 500 mM NH_4_Cl, 0.5 mM EDTA, and 6 mM 2-mercaptoethanol) and spun at 44,000 rpm for 16 h in a Beckman ultracentrifuge 90Ti rotor at 4ºC. The ribosome pellets were washed once with buffer C (50 mM Tris-Cl at pH 7.5, 10 mM MgCl_2_, 100 mM NH_4_Cl, and 6 mM 2-mercaptoethanol) and then resuspended in the same buffer by gentle rocking at 4ºC.

**Ribosome Profile Analysis**

Purified ribosomes were analyzed in 15%-50% (w/v) sucrose gradients in buffer C (50 mM Tris-Cl at pH 7.5, 10 mM MgCl_2_, 100 mM NH_4_Cl, and 6 mM 2-mercaptoethanol). This amount of MgCl_2_ favors ribosomal subunits association. In contrast, to completely dissociate the 70S ribosomes into the individual 30S and 50S subunits, ribosomes were loaded onto 10%-30% (w/v) sucrose gradients in buffer C containing only 0.1 mM MgCl_2_. In both conditions, samples were centrifuged in a Beckman ultracentrifuge SW28 rotor for 16 h at 24,000 rpm at 4ºC. Fractions (1mL) were collected from the top and quantified by A254 measurement on a Nanodrop machine.

For identification of the rRNA species present in the ribosomal fractions, RNA was extracted by phenol:chloroform, precipitated in ethanol and 300 mM sodium acetate in the presence of glycogen as carrier and analyzed on agarose gels stained with ethidium bromide. Proteins in the ribosomal fractions were precipitated by addition of trichloroacetic acid to a final concentration of 10% and washed with cold acetone. Proteins were quantified by the Lowry assay. The localization of RNase R and Hfq was analyzed by Western blot using either RNase R or Hfq polyclonal antibodies.

**Protein purification**

Purification of His_6_-RNase and His_6_-Hfq was performed by histidine-affinity chromatography using HiTrap Chelating HP columns (GE Healthcare) and AKTA FLPC system (GE Healthcare) following the protocols described previously (5, 6). Plasmids pABA-RNR (for overexpressing His_6_-RNase R) and pTE607 (for overexpressing His_6_-Hfq) were transformed into BL21(DE3) *E. coli* strain. Cells were grown at 37°C in LB medium supplemented with 100 μg/ml ampicillin to an OD600 of 0.5 and then protein overexpression was induced by addition of 1 mM IPTG (isopropyl β-D-thiogalactoside) for 3h. Cell cultures were pelleted by centrifugation at 8,500*g* for 15 min and stored at −80 C until use.

For RNase R purification, the cell pellet was resuspended in lysis buffer (50mM Tris at pH 7.5, 100 mM NaCl, 1mM EDTA, 5% glycerol and 1mM DTT) in the presence of Complete Mini Protease Inhibitor Cocktail EDTA-free (Roche) and disrupted by French Press. The crude extracts were treated with Benzonase (Sigma) to degrade the nucleic acids and clarified by a 30 min of centrifugation at 10,000*g*. The clarified extracts were then added to a 1 ml HiTrap Chelating Sepharose column equilibrated in buffer RNR-A (20 mM Tris at pH 8 and 500 mM NaCl) plus 20 mM imidazole and 2 mM 2-mercaptoethanol). Protein elution was achieved by a continuous imidazole gradient (from 20 to 500 mM) in buffer RNR-A. The fractions containing the purified protein were pooled and buffer-exchanged to buffer RNR-B (20 mM Tris at pH 8, 300 mM KCl and 2 mM 2-mercaptoethanol) using a 5 ml desalting column (GE Healthcare). Eluted proteins were concentrated by centrifugation at 7,000*g* for 15 min at 15°C with Amicon Ultra Centrifugal Filter Devices of 30 kDa molecular-mass cut-off (Millipore). RNase R concentration was determined by spectrophotometry measurement at A280 and 50% (v/v) glycerol was added to the final fractions before storage at −20°C.

For Hfq purification, the cell pellet was resuspended in lysis buffer (20mM Tris at pH 7.8, 500mM NaCl, 10% glycerol and 0.1% Triton X‐100) in the presence of Complete Mini Protease Inhibitor Cocktail EDTA-free (Roche) and disrupted by French Press. The crude extracts were treated with Benzonase (Sigma) to degrade the nucleic acids and clarified by a 30 min centrifugation at 10,000*g*. After a clarification step, imidazole–HCl (pH 7.8) was then added to the supernatant to a final concentration of 1 mM and the suspension was applied to a 1 ml HiTrap Chelating Sepharose column. The resin was then sequentially washed with 15 ml of Buffer Hfq-A (20 mM Tris at pH 7.8, 300 mM NaCl and 20 mM imidazole) and 15 mL of buffer Hfq-B (50 mM sodium phosphate at pH 6.0, 300 mM NaCl). Hfq was eluted with buffer Hfq-B with 250 mM imidazole. Fractions containing Hfq were determined by SDS–PAGE analysis, pooled and heated to 80°C for 15 min. Insoluble material was removed by centrifugation and the supernatant was buffer-exchanged to buffer Hfq-C (50 mM Tris–HCl at pH 7.5, 1 mM EDTA, 50 mM NH4Cl, 5% glycerol and 0.1% Triton X‐100) using a 5 ml desalting column (GE Healthcare). Eluted Hfq was concentrated by centrifugation at 7,000*g* for 15 min at 15°C with Amicon Ultra Centrifugal Filter Devices of 3 kDa molecular-mass cut-off (Millipore) and its concentration was determined by spectrophotometric measurement at A280. The protein was kept at 4°C.

**Pulldown assay**

Purified His_6_-RNase R or His_6_-Hfq were incubated with Ni-NTA beads (Qiagen) in 1 mL of binding buffer (50 mM Tris at pH 7.6, 100 mM NaCl, 10 mM imidazole) for 60 min at 4ºC. Stationary phase cultures resuspended in lysis buffer (50 mM Tris at pH 8, 125 mM NaCl, 10% glycerol, 0.1% TritonX-100, 1 mM PMSF) were disrupted by French Press and 1 mg of cell lysates was added to the beads. Incubation proceeded overnight at 4ºC with gentle rocking. The Ni-NTA resin was recovered by centrifugation and washed five times with binding buffer. Bound proteins were then eluted with elution buffer (50 mM Tris at pH 7.6, 100 mM NaCl, 300 mM imidazole). Eluted proteins were separated by SDS-PAGE and probed with Hfq or RNase R antibodies.

**Co-Immunoprecipitation RNase R and Hfq**

Cell lysates were incubated with RNase R antibody bound to Protein A/G agarose beads, based on the instructions of the Pierce Crosslink Immunoprecipitation Kit (Thermo Scientific). Stationary phase cells were disrupted in ice cold IP Lysis (50 mM Tris at pH 8, 500 mM NaCl, 0.1% TritonX-100, 10% glycerol) in the presence of Complete Mini Protease Inhibitor Cocktail EDTA-free (Roche) using the French Press. Protein concentration in cell extracts was determined by Bradford assay. RNase R antibody was bound to Protein A/G Plus beads. 20µl of Protein A/G Plus resin was prepared and washed with Coupling Buffer (10 mM sodium phosphate at pH 7.2, 150 mM NaCl). RNase R antibody was then added to the resin and incubated in a mixer for 2 h at 4ºC. Subsequently, the resin was washed two times with Coupling Buffer. RNase R antibody was further crosslinked to Protein A/G Plus beads through the action of DSS (disuccinimidyl suberate) for 1h at room temperature. The resin was washed twice with Elution Buffer provided with the kit (pH 2.8) and washed two more times with Crosslink Washing Buffer (25 mM Tris at pH 7.4, 15 mM NaCl, 1 mM EDTA, 1% NP-40, 5% glycerol) to eliminate non-crosslinked antibody. 3 mg of cell extracts were added to the antibody-crosslinked resin and incubated overnight at 4ºC with gentle mixing. After centrifugation of the flowthrough, the column was washed three times with TBS (0.02 M Tris, 0.137 M NaCl) and once with Conditioning Buffer provided with the IP kit. Antigens were eluted with sample loading buffer (50 mM Tris at pH 6.8, 2% SDS, 20 mM DTT, 10% glycerol, 0.04% BB) following a 10 min incubation at 100ºC. Samples were collected by centrifugation and eluted Hfq in complex with RNase R was identified by Western blot analysis

**Far-Western blot analysis**

Far-Western blotting was performed as described previously (7). RNase R and Hfq were purified as described above. Benzonase (Sigma-Aldrich) was used for elimination of contaminating nucleic acid during the protein extraction. Increasing amounts of purified RNase R were loaded on a SDS-PAGE gel and transferred to a nitrocellulose membrane (Hybond ECL, GE Healthcare). BSA was used as a control. The membrane was stained with Ponceau S to verify the presence of immobilized proteins. After destaining, the proteins in the membrane were subject to a denaturation process in freshly prepared AC buffer (100 mM NaCl, 20 mM Tris pH 7.5, 0.5 mM EDTA, 10% glycerol, 0.1% Tween-20, 2% skim milk powder and 1 mM DTT) containing decreasing amounts of guanidine-HCl from 6 to 0.1 M. Renaturation of RNase R was done overnight at 4°C in AC buffer without guanidine-HCl. Membranes were blocked with 5% milk in TBST (20mM Tris at pH 7.6, 137 mM NaCl, 0.1% Tween-20) for 1 h at room temperature. Purified Hfq was added to AC buffer at a final concentration of 1 µg/ml and membranes were incubated overnight at 4°C. Membranes were washed three times in TBST and incubated with primary (anti-Hfq) and secondary (horseradish-conjugated) antibodies in TBST with 5% milk. After incubation in Western Lightning Plus-ECL (Perkin-Elmer), the chemiluminescent signal was detected by the Chemidoc XRS^+^ system (Bio-Rad).

**Supplemental References**

1. Leong V, Kent M, Jomaa A, Ortega J. 2013. *Escherichia coli* *rim*M and *yje*Q null strains accumulate immature 30S subunits of similar structure and protein complement. RNA 19:789–802.

2. Charollais J, Pflieger D, Vinh J, Dreyfus M, Iost I. 2003. The DEAD-box RNA helicase SrmB is involved in the assembly of 50S ribosomal subunits in *Escherichia coli*. Mol Microbiol 48:1253–65.

3. Andrade JM, Pobre V, Matos AM, Arraiano CM. 2012. The crucial role of PNPase in the degradation of small RNAs that are not associated with Hfq. RNA 18:844–55.

4. Zundel MA, Basturea GN, Deutscher MP. 2009. Initiation of ribosome degradation during starvation in *Escherichia coli*. RNA 15:977–83.

5. Matos RG, Barbas A, Arraiano CM. 2009. RNase R mutants elucidate the catalysis of structured RNA: RNA-binding domains select the RNAs targeted for degradation. Biochem J 423:291–301.

6. Folichon M, Arluison V, Pellegrini O, Huntzinger E, Régnier P, Hajnsdorf E. 2003. The poly(A) binding protein Hfq protects RNA from RNase E and exoribonucleolytic degradation. Nucleic Acids Res 31:7302–10.

7. Wu Y, Li Q, Chen X-Z. 2007. Detecting protein-protein interactions by Far western blotting. Nat Protoc 2:3278–3284.
